# Comparing Aerodynamic Efficiency in Birds and Bats Suggests Better Flight Performance in Birds

**DOI:** 10.1371/journal.pone.0037335

**Published:** 2012-05-18

**Authors:** Florian T. Muijres, L. Christoffer Johansson, Melissa S. Bowlin, York Winter, Anders Hedenström

**Affiliations:** 1 Department of Biology, Lund University, Lund, Sweden; 2 Department of Biology, University of Washington, Seattle, Washington, United States of America; 3 Department of Natural Sciences, University of Michigan-Dearborn, Dearborn, Michigan, United States of America; 4 Cognitive Neurobiology, Humboldt University and NeuroCure Center of Excellence at the Charité Universitätsmedizin Berlin, Berlin, Germany; University of Hull, United Kingdom

## Abstract

Flight is one of the energetically most costly activities in the animal kingdom, suggesting that natural selection should work to optimize flight performance. The similar size and flight speed of birds and bats may therefore suggest convergent aerodynamic performance; alternatively, flight performance could be restricted by phylogenetic constraints. We test which of these scenarios fit to two measures of aerodynamic flight efficiency in two passerine bird species and two New World leaf-nosed bat species. Using time-resolved particle image velocimetry measurements of the wake of the animals flying in a wind tunnel, we derived the span efficiency, a metric for the efficiency of generating lift, and the lift-to-drag ratio, a metric for mechanical energetic flight efficiency. We show that the birds significantly outperform the bats in both metrics, which we ascribe to variation in aerodynamic function of body and wing upstroke: Bird bodies generated relatively more lift than bat bodies, resulting in a more uniform spanwise lift distribution and higher span efficiency. A likely explanation would be that the bat ears and nose leaf, associated with echolocation, disturb the flow over the body. During the upstroke, the birds retract their wings to make them aerodynamically inactive, while the membranous bat wings generate thrust and negative lift. Despite the differences in performance, the wake morphology of both birds and bats resemble the optimal wake for their respective lift-to-drag ratio regimes. This suggests that evolution has optimized performance relative to the respective conditions of birds and bats, but that maximum performance is possibly limited by phylogenetic constraints. Although ecological differences between birds and bats are subjected to many conspiring variables, the different aerodynamic flight efficiency for the bird and bat species studied here may help explain why birds typically fly faster, migrate more frequently and migrate longer distances than bats.

## Introduction

The independent evolution of powered flight in birds and bats begs the question of whether the apparent convergence in size, shape and flight style has resulted in the same overall flight performance, or if they differ in any aspect. Most birds and bats operate in the same Reynolds number regime (*Re = Uc/ν*∼10^4^, where *U* is the flight speed, *c* is the wing chord length and *ν* is the kinematic viscosity of air) [1], [2], which indicates an overall fluid dynamic similarity [3]. Thus, any difference in flight performance would be due to variation in ecological requirements or phylogenetic constraints on morphology [4], [5]. For example, night-active bats have protruding ears required for echolocation, which affects the drag generated by the body [6]. Bats have wings formed by skin membranes stretched between elongated finger bones, while bird wing surfaces are formed of adjacent feathers radiating from reduced skeleton bones. To date, there are a few studies that suggest possible differences in flight efficiency between birds and bats, but with conflicting results [7]–[9]. Thus, the available data on flight performance in birds and bats are too limited to draw any general conclusions on differences in relative flight performance as a result of differences in phylogeny.

In the present study, we studied the effect of phylogenetic origin of flight on performance by comparing the aerodynamic flight performance for two passerine bird species and two New World leaf-nosed bat species, flying across a range of flight speeds under similar conditions in a wind tunnel [10], [11]. Flight performance was measured by studying the aerodynamic wake produced by the flying animals using time-resolved particle image velocimetry (PIV) [11]–[13]. Since these types of studies are relatively time consuming and labor intensive, the number of species that can realistically be studied is limited. Therefore, it is important to select the study species as carefully as possible such that a bias due to differences in ecology is reduced. We studied three pied flycatchers (*Ficedula hypoleuca*) (body mass 14 g), one blackcap (*Sylvia atricapilla*) (16 g), two Pallas' long-tongued bats (*Glossophaga soricina*) (10 g), and two lesser long-nosed bats (*Leptonycteris yerbabuenae*) (22 g), Table 1. The flight dynamics data for each species that this study was based on are reported elsewhere [12]–[15]. The species studied are similar in size and thus fly at similar *Re* (∼10^4^), which might lead to similar aerodynamic performance [1]–[3]. The selected bird and bat species also partly overlap in feeding and migration behavior, which are ecological factors potentially influencing flight performance [2] (see Discussion for more detail). For the species studied, we determined two independent quantitative measures of the relative flight performance: the span efficiency, a measure for the efficiency of lift production [13], [16], [17] and the effective lift-to-drag ratio for flapping flight, a metric associated with energetic flight efficiency [12], [18]. The results were compared among the species, and differences in performance between the birds and bats were related to differences in morphology, kinematics, ecology and fluid dynamics, to identify the effect of phylogenetic origin of flight on the performance of vertebrate flight.

**Table 1:** Morphological data for the experimental animals.

*species (gender)*	*M*	*b*	*S*	*c*	*AR*	*Q*
	*(kg)*	*(m)*	*(m^2^)*	*(m)*	*(−)*	*(N/m^2^)*
*F. hypoleuca #1*	*0.0148*	*0.235*	*0.0106*	*0.045*	*5.2*	*13.7*
*F. hypoleuca #2*	*0.0141*	*0.235*	*0.0105*	*0.045*	*5.3*	*13.2*
*F. hypoleuca #3*	*0.0137*	*0.236*	*0.0107*	*0.045*	*5.2*	*12.6*
*S. atricapilla*	*0.0163*	*0.240*	*0.0111*	*0.046*	*5.2*	*14.4*
*G. soricina (Male)*	*0.0101*	*0.233*	*0.00879*	*0.038*	*6.2*	*11.3*
*G. soricina (Female)*	*0.0095*	*0.230*	*0.00860*	*0.037*	*6.2*	*10.8*
*L. yerbabuenae (Male)*	*0.0216*	*0.335*	*0.01576*	*0.047*	*7.1*	*13.4*
*L. yerbabuenae (Female)*	*0.0236*	*0.323*	*0.01529*	*0.047*	*6.8*	*15.1*

Mass *M*, wingspan *b*, wing surface area *S*, mean chord length *c = S/b*, aspect ratio *AR = b^2^/S* and wing loading *Q = Mg/S*, where *g* is gravity.

## Materials and Methods

### Ethics statement

The experiments were carried out in accordance with university guidelines and approved by the Malmö/Lund Animal Research Ethics Committee of the counties of Blekinge, Skåne and Halland (Malmö/Lunds Djurförsöksetiska Nämnden, Blekinge, Skåne och Hallands län).

### Experimental animals

Three pied flycatchers, one blackcap, two Pallas' long-tongued bats, and two lesser long-nosed bats were used for the experiments. Morphological data for all experimental animals are in Table 1.

### Experiments

The experimental setup consisted of the Lund University low-turbulence, low-speed wind tunnel [10], [19], a high-speed (200 Hz) stereo Particle Image Velocimetry system (PIV), and two high-speed kinematics cameras running at 250 Hz (Fig. S1) [11]. For the nectar-feeding bats, we used a honey-water feeder to position them in the tunnel. When a bat was flying steadily at the feeder, we sampled the wake behind the animal using the PIV system. The birds were trained to perch in the test-section of the wind tunnel. When the perch was lowered, the bird took off. If the bird flew steadily in the correct position, PIV measurements were performed, after which the perch was presented again. The temperature ranged between 20–25°C during experiments.

Using this experimental procedure, we did experiments at a wind tunnel speed range of *U* = 2–9 m/s, in increments of 1 m/s. For the pied flycatchers, measurements were done for flycatcher #3 at 2, 4, and 7 m/s and for flycatcher #1 and #2 at all flight speeds between *U* = 2–7 m/s. For the blackcap, measurements were done at *U* = 7, 8 and 9 m/s. For the bats, PIV was measured at all flight speeds from 2 m/s to 7 m/s. For each measured individual-speed combination, PIV data of at least 10 wingbeats was analyzed.

### PIV analysis

The stereo PIV data was analyzed using DaVis (LaVision, DaVis 7.2.2.110), resulting in three-dimensional velocity vectors {*u,v,w*} within each node point {*y,z*} in each PIV frame [11]. The PIV frames within one wingbeat were given a frame number *n* = [1−*N*] (*n* = 1 for the beginning of the downstroke and *n = N* at the end of the upstroke), a non-dimensional time stamp *τ* = [0–1], and a streamwise position *x* = [0−*λ*] (*λ* is the wingbeat wavelength). The non-dimensional time stamps are defined as *τ = t/Π*, where *Π* is wingbeat period. *t* is the timing within the wingbeat where *t* = 0 corresponds to the start of the downstroke, *t* = *Π* is at the end of the upstroke, and *t* = *R_ds_Π* is at the end of the downstroke, where *R_ds_* is the wingbeat downstroke ratio. For the frames in between, *t* was linearly interpolated. Assuming that the wake convects statically downstream with the forward flight speed, the streamwise position of each PIV frame is *x = (n−1)UΔt*, where *Δt* is the inverse of the PIV frame rate (1/200 Hz) [12], [15].

The PIV results were analyzed by identifying the main vortices in the wake: the tip vortex, root vortex, tail vortex and reversed vortex loops [14], [20]. In each PIV frame, the position {*x,y,z*} and circulation *Γ* of the present vortex structures were measured using a custom-made Matlab (7.7.0.471, R2008b) PIV analysis program [12], [15]. From this, the resultant normalized aerodynamic lift of each vortex structure was calculated using basic vortex theory [3], [21], [22] as
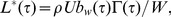
(1)where *W* is the weight of the animal, *ρ* is the air density and *b_w_(τ)* is the wake span determined from the *y*-position of the vortex structure (Fig. S2A) [15]. The normalized thrust component of the aerodynamic force of each vortex structure is determined by
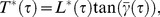
(2)where 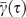 is the mean streamwise vortex system angle. For the tip vortex system, it is determined by
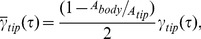
(3)where *γ_tip_ (τ)* is the spanwise tip vortex angle (Fig. S2) [12], [15]. *A_tip_* and *A_body_* are the vertical wingbeat amplitude of the wingtip and body, respectively, determined from kinematics measurements [12], [14], [15], [23], [24]. There is a minus sign in front of *A_body_*/*A_tip_* because the vertical body movement is in anti-phase with the wing movement (body moves up when wings move down). For the root vortex system, it is determined by
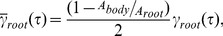
(4)where *A_body_*/*A_root_* is estimated *A_body_*/*A_root_ = A_body_*/*A_tip_·A_tip_*/*A_root_*, and *A_tip_*/*A_root_* is determined from the relative movement of tip and root vortex. For the reversed vortex loop and tail vortex we assume 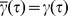 [12], [15].

The vertical induced velocity distribution w(y^*^) along the normalized span y^*^ was measured in each PIV frame along a straight line from the position behind the animal's body center line to the spanwise most distal vortex structure [13]. The non-dimensional span is defined as y^*^ = y/b, where *b* is the wingspan.

### Average wingbeats

We determined the average wingbeat for a certain species-speed combination by averaging the results of all measured wingbeats for that species-speed combination, using smoothing splines. The average wingbeat wake consists of the average temporal normalized lift 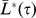 and thrust 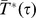 of all vortex structures [12], [15] and the average spanwise and temporal downwash distribution 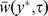 [13], for each species-speed combination. The relative variation in the spanwise downwash distribution 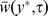 was estimated by determining a sliding 95% confidence interval of *m* local data points, with *m* the amount of analyzed wingbeats [13].

### Aerodynamic forces and performance

By integrating 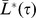 and 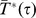 throughout the wingbeat we determined the wingbeat average lift-to-weight ratio *L/W*, the effective lift-to-drag ratio *L/D* (*D* = *T* for steady flight [12]), and the normalized cost-of transport *COT* = 1/(*L/D*). By integrating 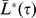 and 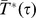 throughout the downstroke and upstroke we determined *L/W_down_*, *T/W_down_*, *L/W_up_*, and *T/W_up_*, respectively. From the average spanwise and temporal downwash distribution 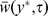, the real induced power *P_i_* and ideal induced power *P_i ideal_* were estimated. *P_i_* is the induced power for generating *L* and *P_i ideal_* is the minimum induced power required to generate *L*, based on the equivalent uniform spanwise downwash. The effective span efficiency for a complete wingbeat is defined as *e_i_* = *P_i ideal_*/*P_i_* [13], [17].

### Statistical analysis

To control for differences in weight between species, which is expected to affect the characteristic flight speed according to *W* ^1/6^ [18], [25], we used normalized flight speeds for the statistical analyses. The normalized flight speed for a certain individual is defined as *U* ^*^ = *U* (*W*_ref_/*W*)^1/6^, where *W* is the weight of the individual and *W*_ref_ is the average weight of the Pallas' long-tongued bats [12]. The results for the different animals were compared using mixed linear models. *L/D*; *COT*; *e_i_*; *L/W_down_*; *T/W_down_*; *L/W_up_* or *T/W_up_* was set as the dependent variable. For all tests *Bird/Bat*; U^*^; U^*^×Bird/Bat were set as factors, while only for *L/D*, and *COT* we added (*U* ^*^)^2^; (*U* ^*^)^2^ *×Bird/Bat* as factors. *Species nested within Bird/Bat* was set as a random variable.

## Results

**Figure 1:**
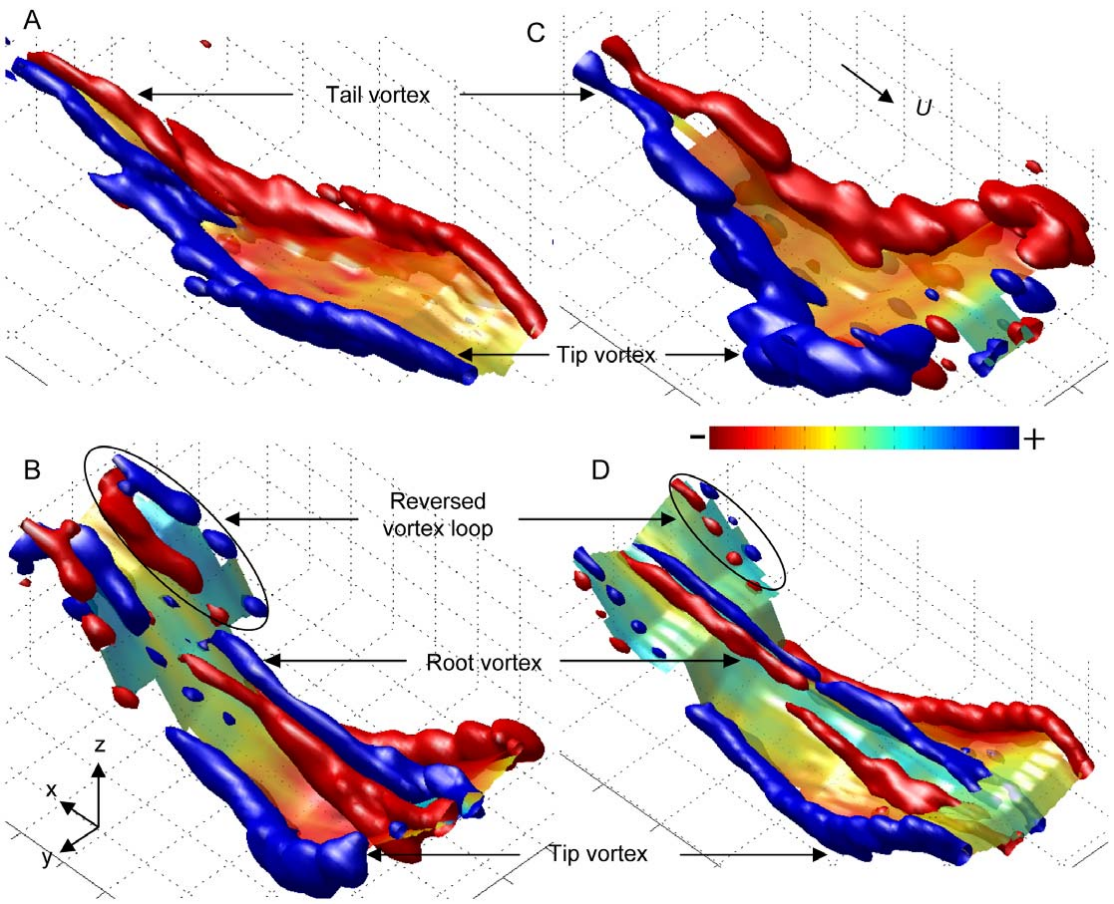
Wake topologies for one wingbeat of the studied species flying at 7 m/s. The vorticity iso-surfaces (blue: +*ω_x_*_ iso_; red: −*ω_x_*_ iso_) show the main vortex structures, while the color-coded surface shows downwash *w* (see color bar). (A) Wake of pied flycatcher #1, *ω_x_*_ iso_ = ±50 s^−1^ and *w_max_* = 1.7 m/s; (B) female Pallas' long-tongued bat with *ω_x_*_ iso_ = ±50 s^−1^ and downwash scale *w_max_* = 2.1 m/s; (C) the blackcap, *ω_x_*_ iso_ = ±70 s^−1^ and *w_max_* = 3.0 m/s; (D) female lesser long-nosed bat, *ω_x_*_ iso_ = ±45 s^−1^ and *w_max_* = 2.4 m/s. The wind tunnel coordinate system {*x,y,z*} and freestream velocity vector *U* are in panel (B) and (C), respectively.

**Figure 2:**
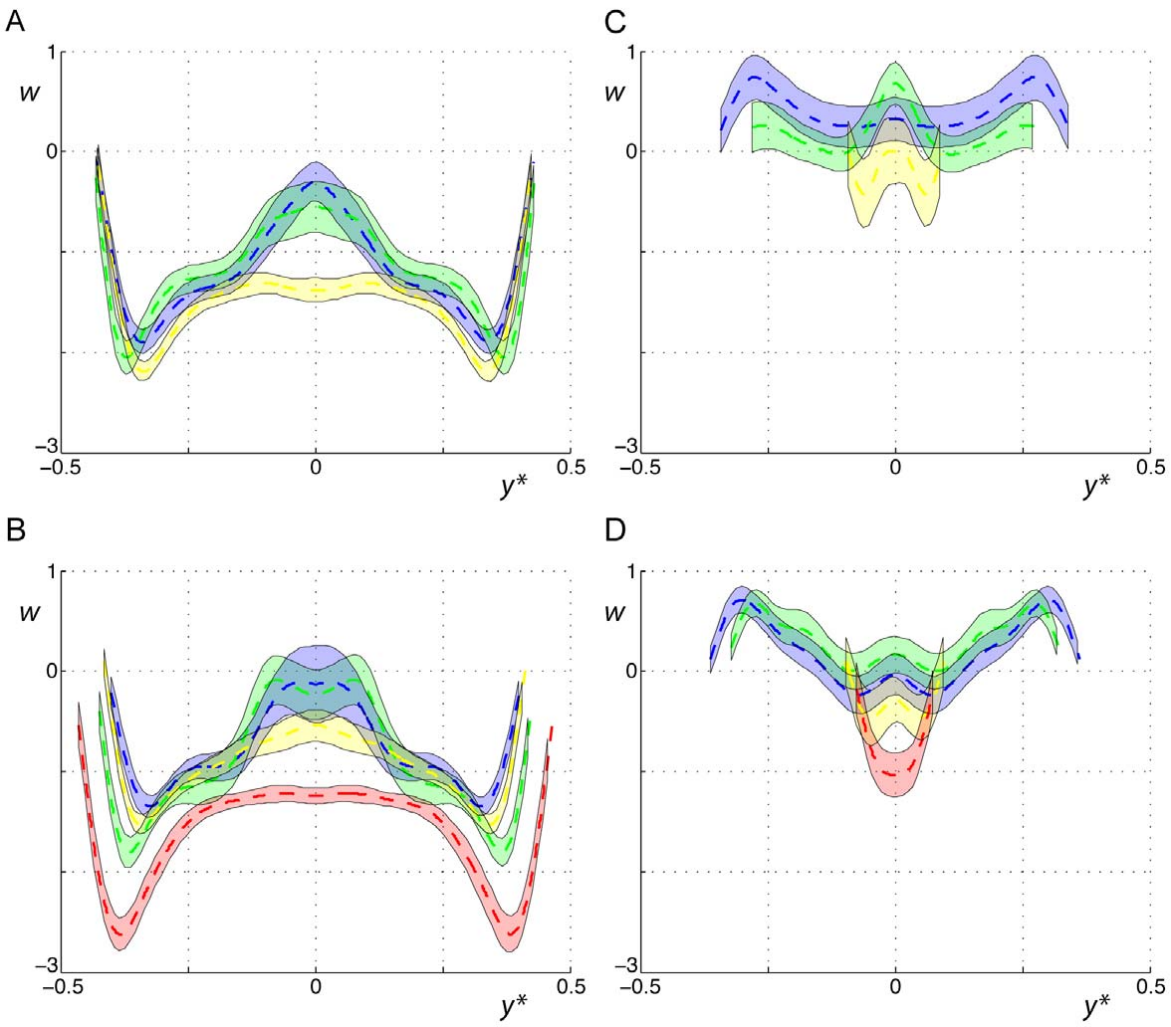
Spanwise downwash distributions at mid-downstroke and mid-upstroke for the studied species. Spanwise downwash at mid-downstroke at 4 m/s (A) and 7 m/s (B) and mid-upstroke at 4 m/s (C) and 7 m/s (D), for the pied flycatcher (yellow), blackcap (red), Pallas' long-tongued bat (blue), and lesser long-nosed bat (green). The dotted lines show the average downwash for all measurements and the bars around the dotted lines are the sliding 95% confidence interval.

The aerodynamic wakes generated by a representative wingbeat were visualized for the different species studied flying at *U* = 7 m/s (Fig. 1 and Fig. S3, S4, S5, S6). For both the birds and bats, each wing generates a wing ‘tip vortex’ and a wing ‘root vortex’ at the start of the downstroke. For the birds, the root vortices disappear immediately after the start of the downstroke, while for the bats the root vortices are present throughout the complete wingbeat. The stronger root vortices during the downstroke in the bats result in a reduction in downwash behind the body compared to the birds (Fig. 2A–B). The tip vortices are present throughout the complete downstroke, but disappear during the upstroke. For the birds, the tip vortices consistently disappear earlier within the upstroke than for the bats (Fig. S7A–B). For the birds, but not the bats, a new vortex pair appears closer to the body about the same time as when the tip vortex disappears (Fig. 1). We assume that these are shed from the body/tail configuration, so they are labeled ‘tail vortices’ [14], [15], [26], [27]. The tail vortices are present until the end of the upstroke and add to lift (Fig. S7A–B) [15]. The bats, but not the birds, generate a vortex loop behind each wing during the latter part of the upstroke (Fig. 1) [11]–[13], [20], [28]–[30]. These vortex loops result in a negative lift signified by an upwash at the outer wing (Fig. 2C–D, Fig. S7C–D), and are therefore labeled ‘reversed vortex loops’ [12]. Thus, the bats generate a more complex wake, including stronger root vortices and reversed vortex loops, than the birds [20]. For more details on the wake structure we refer to previous studies of the wake dynamics for these species [12]–[15], [20].

**Figure 3:**
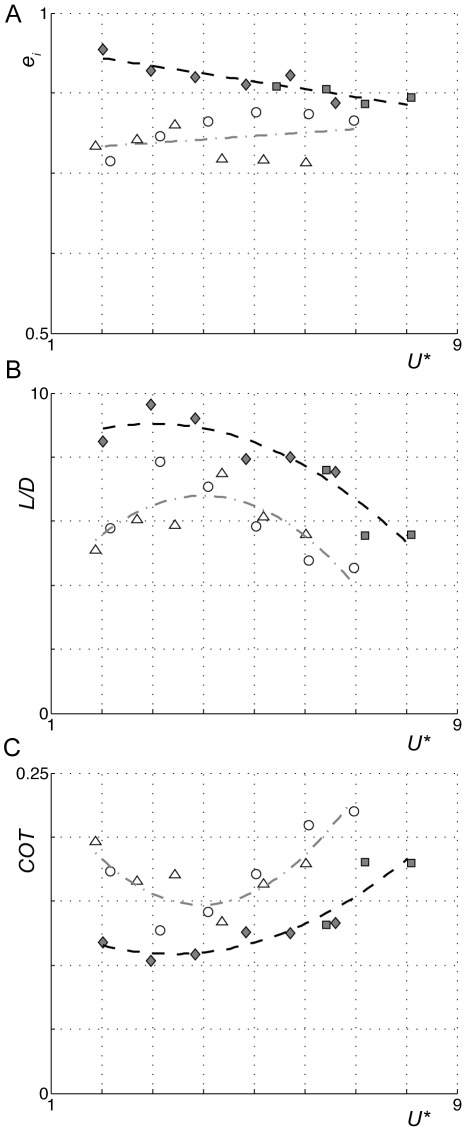
Flight efficiency factors throughout the measured normalized flight speed range for the studied species. The different metrics are span efficiency *e_i_* (A); effective lift-to-drag ratio *L/D* (B); and the normalized mechanical cost-of-transport *COT* (C), for the pied flycatcher (filled diamonds), blackcap (filled squares), Pallas' long-tongued bat (open circles), and lesser long-nosed bat (open triangles). The trend lines are for birds (black dash) and bats (grey dot dash).

The wingbeat average lift-to-weight ratio across the measured flight speed range was *L/W* = 0.99±0.03 (mean±standard deviation) for the Pallas' long-tongued bat; *L/W* = 0.95±0.04 for the lesser long-nosed bat; *L/W* = 0.93±0.10 for the pied flycatcher; and *L/W* = 1.14±0.11 for the blackcap. The two independent aerodynamic performance metrics, the lift-to-drag ratio *L/D* and span efficiency *e_i_* were significantly higher for the birds compared to the bats, throughout the complete measured flight speed range (*p* = 0.0088 and *p*<0.0001, respectively, Table 2; Fig. 3A–B).

**Table 2:** Statistical results for the mixed linear model analysis of lift-to-drag ratio *L/D*, cost-of-transport *COT* and span efficiency *e_i_*.

		*L/D*			*COT*			*e_i_*	
	*DF*	*F-ratio*	*r^2^*	*DF*	*F-ratio*	*r^2^*	*DF*	*F-ratio*	*r^2^*
*Overall Model*	*7*	*11.17*	*0.8574*	*7*	*11.86*	*0.8647*	*5*	*18.53*	*0.8607*

	*DF*	*t-ratio*	*p-value*	*DF*	*t-ratio*	*p-value*	*DF*	*t-ratio*	*p-value*
*Intercept*	*-*	*14.07*	***<0.0001***	*-*	*5.55*	***<0.0001***	*-*	*44.29*	***<0.0001***
*Bird/Bat*	*1*	*−3.08*	***0.0088***	*1*	*2.54*	***0.0248***	*1*	*−7.82*	***<0.0001***
*U*	*1*	*−4.06*	***0.0014***	*1*	*4.34*	***0.0008***	*1*	*−1.56*	*0.1401*
*U×Bird/Bat*	*1*	*−0.10*	*0.9194*	*1*	*1.31*	*0.2131*	*1*	*2.14*	***0.0490***
*U^2^*	*1*	*−3.76*	***0.0024***	*1*	*4.64*	***0.0005***	*-*	*-*	*-*
*U^2^×Bird/Bat*	*1*	*−1.58*	*0.1390*	*1*	*2.41*	***0.0312***	*-*	*-*	*-*

Variables are the degrees-of-freedom (*DF*), *F*-ratio, the *r^2^*-value, *t*-ratio, and *p*-values. The *p*-values in bold are significant.

To test how the difference in *L/D* for the bats and the birds affects the flight dynamics, we consider the normalized lift and thrust production during the downstroke and upstroke separately (*L/W_down_*, *T/W_down_*, *L/W_up_* and *T/W_up_*, respectively; Fig. S8). Note that, for steady flight, the mean thrust throughout the wingbeat is equal to the mean total drag *D* [12]. During the downstroke, both lift and thrust production increased with flight speed (Fig. S8A–B), similarly for the birds and bats (*p* = 0.9954 and *p* = 0.1266 for *L/W_down_* and *T/W_down_*, respectively; Table S1). During the upstroke, however, the lift and thrust production scale very differently with flight speed in the birds and bats (*p* = 0.0002 and *p*<0.0001 for *L/W_up_* and *T/W_up_*, respectively; Table S2 and Fig. S8C–D). *L/W_up_* decreases with flight speed for the bats, while it increases for the birds, and *T/W_up_* is close to zero for the birds, while it varies significantly with flight speed for the bats.

The normalized mechanical cost-of-transport *COT* is a non-dimensional variable that represents the amount of mechanical energy required to transport a unit weight across a unit distance (*COT = E/(Wd)*, where *E* is energy required and *d* is distance) [18], [31]. For flying animals, the cost-of-transport can be estimated by *COT* = 1/(*L/D*). The *COT* distribution across flight speeds shows the familiar u-shaped curve for both the birds and the bats (Fig. 3C), but the values for the bats are significantly higher than for the birds (*p* = 0.0248, Table 2). We estimated migratory flight performance based on the average *COT* distributions for bird and bat, by assuming equal energy available for mechanical work in the birds and bats. We analyzed two scenarios: (1) If a bird and bat fly at the same flight speed of *U* ^*^ = 6 m/s, the bird can fly 28% further than an equally sized bat (*d_bird_* = 1.4 *d_bat_*, Fig. 3C). (2) If the migration distance is the same for a bird and a bat and the bat operates at minimum *COT* (*COT* = 0.15 at U^*^ = 4.0 m/s, Fig. 3C), the bird can fly 41% faster than the bat (U^*^ = 6.7 m/s for the bird, Fig. 3C).

## Discussion

The energetic cost of flight is directly related to the amount of drag produced by a flying animal [1]. This drag can be divided into drag produced by the wing (profile drag), drag produced by the body (parasite drag), and drag resulting from the downwash generated behind the animal (induced drag). The span efficiency *e_i_* is related to the induced drag, since it is a measure of the deviation from a constant spanwise downwash distribution associated with an elliptic lift distribution, which results in minimum induced drag (*e_i_* = 1) [13], [17]. Our bats deviated more from a constant spanwise downwash distribution than our birds (Fig. 2), mainly due to reduced downwash behind the body (Fig. 2A–C), which is a result of lower body lift in the bats [13]. Thus, since our bats generate less body lift than our birds, their span efficiency is lower (Fig. 3A) and consequently also *L/D* is lower [3], [17]. The fact that the bats generate less body lift could be a result of having less streamlined bodies than the birds, e.g. due to the presence of the protruding nose leaf and ears required for echolocation [5], [7], [32]. The protruding ears can be expected to also increase the parasite drag, as was found in a model of a brown long-eared bat (*Plecotus auritus*) [6], and since concave shapes such as these ears are known to be the most drag producing bluff bodies [33].

The differences in wake dynamics between the birds and bats during the upstroke are mainly a result of the presence of reversed vortex loops in the bats and tail vortices in the birds (Fig. 1). The reversed vortex loops in the bats are generated by moving the wing upwards at a negative angle-of-attack resulting in production of positive thrust and negative lift [12], [20], [28]. With increasing flight speed this negative lift and positive thrust also increase (Fig. S8C–D) [12]. The birds, on the other hand, make their wings inactive (feathered) during the latter part of the upstroke by retracting them and by spreading the primary wing feathers [14], [15], [34], [35]. Therefore the birds primarily generate body lift during the latter part of the upstroke, resulting in tail vortices (Fig. S8C–D) [14], [15], [26]. Hence, there is a clear qualitative and quantitative difference in the function of the upstroke between the passerine birds and the leaf-nosed bats studied here, which could be directly related to the difference in *L/D*.

Hall and colleagues [36], [37] modeled flight efficiency in large-amplitude flapping wing configurations relevant for flapping flight of birds and bats. They found that for flapping wings with relatively low *L/D* (*L/D* = 5, i.e. similar to that of the bats), the energetically optimal flapping kinematics generate thrust in combination with negative lift during the upstroke (i.e. resulting in reversed vortex loops) [36]. Achieving this wake thus require wing kinematics to have negative angles-of-attack of the outer wing during the upstroke. For configurations with higher *L/D* (*L/D* = 10, i.e. similar to that of the birds) the upstroke should optimally generate positive lift [36]. So, both the birds and bats have wake topologies that are near the optima for the respective *L/D* regime they operate at [36], [37].

The lift-to-weight ratios determined from the vortex wake dynamics are close to one, and hence we can assume that the majority of the wake dynamics forces are captured (Fig. S5), resulting in a realistic estimate of *L/D* and *COT*. The fact that *L/W* for the blackcap was on average higher than one, can be explained by the fact that the blackcap sporadically bounded during the experiments (as they also naturally do), particularly at the highest measured flight speed. To compensate for the low lift produced during these bounds the bird enhanced its lift during the flapping phases, resulting in *L/W*>1 [26], [38].

Although the analysis of the results can be considered solid, we caution against over-generalizing the results considering the small number of species in this study (total of eight individuals from four species). However, previous studies have also hinted at similar differences in aerodynamic performance between birds and bats [7], [8], while results based on physiological measurements are less conclusive [8], [9]. The generality of our results are further supported by results from the larger dog-faced fruit bat *Cynopterus brachyotis* (*Megachiroptera*) [29] and the insectivorous *Tadarida brasiliensis* (*Microchiroptera*) [30], which have different flight ecology than the bats used here. The latter species has relatively high aspect ratio wings and feeds on insects in the open airspace. Despite the difference in flight ecology and morphology they have wake patterns strikingly similar to that of our leaf-nosed bats, including the main characteristics responsible for the lower performance of our bats, i.e. the reversed vortex loops during the upstroke and root vortices throughout the wingbeat as a result of relatively low body lift [29], [30]. Additionally, to the best of our knowledge, all kinematics studies of flying bats to date [23], [24], [39]–[43] have found the upstroke wing movement responsible for generating the negative lift (i.e. negative angle-of-attack at the hand wing section) that produces reversed vortex loops [20]. The upstroke wake topology for our passerine birds, consisting of primarily tail vortices, is likewise similar to the upstroke wake found in other birds, including non-passerines (for the longitudinal PIV measurements, spanwise circulation during the upstroke was maximum behind the body suggesting minimum influence of root vortices) [22], [27], [44], [45]. Taken together, these studies suggest that the results presented here are representative for at least New World leaf-nosed bats and passerine birds, and that further testing if the results hold for birds and bats in general should be fruitful.

There is a possibility that other factors than phylogenetic origin could explain the results found here, such as the fact that bats flew behind a feeder, which induces station-holding flight, while the flying birds were only restrained within the test section. However, station-holding flight was also observed in the slow-flying flycatcher. In addition, as mentioned above, the wake structures found in the bats (strong root vortices and reversed vortex loops at the end of the upstroke) have also been found in other species of bats not flying behind a feeder [29], [30].

The wingbeat kinematics differs between birds and bats, which may introduce a bias in the results. However, we argue that the differences in kinematics are directly related to the differences in morphology between our birds and bats rather than being independent characteristics. If, for example, the negative angle-of-attack at the hand wing section found in bats was undesired (since it results in negative lift) the bats could hold their wings at zero angle-of-attack and avoid generating the vortex. However, if, as we argue, generating the vortices is improving performance when *L/D* is relatively low (due to higher induced and parasite drag) [36], [37], the kinematic differences between birds and bats are a direct result of the differences in morphology. Separating the effect of kinematics and morphology is thus difficult since it is the combination of the two that generate the resulting aerodynamics. To further explore the separate effect of kinematics and morphology we suggest studying flight dynamics in mechanical flappers where the kinematics can be altered independent of morphology [46].

Another important question is how much of the variation in flight performance between the birds and bats that was found here is due to differences in ecology. The two ecological factors that are expected to influence flight performance most directly are movement and feeding ecology. With respect to feeding ecology, both bat species are nectarivorous Glossophaginae bats that hover in front of flowers when feeding [32], [47]. The pied flycatcher is an insectivorous passerine that catches insects mostly during short flights involving slow flight and hovering, but also gleans from leaves and twigs [48]. The blackcap primarily finds its food without flying at all, by gleaning insects and berries from leaves and twigs among shrubs [48]. Thus, the foraging niches of the pied flycatcher and the bats are partly overlapping, although the bats fly continuously during foraging while the birds do not. This might result in a slight bias towards higher flight efficiency in the bats compared to the birds, especially at low flight speeds [9]. As this was not supported by our data (Fig. 3), we conclude that feeding ecology cannot explain the difference in flight performance found between our birds and bats.

With respect to movement ecology, the Pallas' long-tongued bat is a residential species with a relatively small home range [47], while the lesser long-nosed bat is migratory [49]. In fact, its migratory route is among the longest found in bats and it is therefore regarded as a long-distance migratory bat species [49]. Additionally, the lesser long-nosed bat commutes every night up to 100 km from its roost site to different feeding patches [50]. The pied flycatcher is a long-distance migratory bird, with its breeding range throughout Europe and wintering grounds in western and central Africa [48]. The blackcap is a partially migratory European passerine, where some individuals migrate and others do not. Among the migrating individuals, some stay within Europe, while others migrate annually to Africa and back [48].

Thus, based on the movement ecology of our birds and bats we may expect a slight bias towards higher flight performance in the birds at high flight speeds. Should this bias be the primary factor for explaining the differences in aerodynamic efficiency between our bats and birds in general, then the wake dynamics and the resulting aerodynamic performance should be more similar among migrants (both birds and bats) than between migrants and non-migrants. This was not supported by our data, as the two studied bat species, although being positioned at least close to the two extremes in the movement ecology landscape for bats (residential species versus a long-distant migrant), have much more similar wake patterns (Fig. 1–2) and maximum flight performance (Fig. 3) than compared to those of the birds. Note that there are significant differences in flight performance between our two bat species that can be related to movement ecology, as the migratory lesser long-nosed bat flies most efficiently (highest *L/D*) at a significantly higher speed than the non-migratory Pallas' long-tongued bat [12], but the maximum performance values (*L/D*) are very similar between the two species (Fig. 3, Table 2), for details see [12]. Thus, the variations in aerodynamic flight performance due to differences in movement ecology, although significant for the bats, are much smaller than the variations found between our birds and bats. Therefore, we conclude that movement ecology is not the main explanatory variable for the difference between our birds and bats. This is further supported by the fact that, as discussed above, kinematics and wake dynamics are more similar among all currently studied birds and bats than between birds and bats [12]–[15], [20], [22]–[24], [26]–[30], [39]–[45], independent of movement ecology.

Taken together, this suggests that our birds and bats may not be able to reach the same level of performance primarily due to phylogenetic constraints [4]. However, to test this hypothesis more thoroughly, we suggest future studies to estimate the aerodynamic flight performance of additional bird and bat species that span larger ecological and morphological ranges, and for additional flight modes such as in gliding and maneuvering flight.

Due to the higher flight efficiency in our birds compared to our bats, the birds have a lower mechanical cost-of-transport *COT* (Fig. 3C). The flight speed at which *COT* is minimum for the bats (U^*^ = 4.0 m/s) is similar to the average natural flight speed of lesser long-nosed bats when commuting over land (U^*^ = 3.7 m/s for *U* = 4.3 m/s) [50], suggesting that the estimated *COT* curves are relevant for predicting optimal flight speeds. However, the difference in mechanical *COT* does not need to be followed by a similar difference in the actual energetic *COT*. There is a possibility that the higher mechanical *COT* in bats could be compensated for by higher energy conversion efficiency in the bats [7], as suggested by the similar power consumption in birds and bats [8], [9].

The independent evolution of flight in birds and bats has resulted in two different wing designs. In this study we show that the feathered avian wing is made inactive during the upstroke and body lift is produced, while the membranous bat wing generates significant flight forces during the upstroke. Both sets of wingbeat kinematics are close to optimal for the relative flight performance regime [36], [37], suggesting that evolution has optimized performance relative to the respective conditions of birds and bats, but that maximum performance is limited by phylogenetic constraints on wing and body morphology [4]. Although optimal migration strategies depend on many conspiring variables [51], [52], the differences in flight performance between the birds and bats found here may help explain why bats typically fly slower, migrate less frequently and shorter distances than birds [32], [52]–[55].

## Supporting Information

Figure S1 **The experimental setup.** It consists of a low-speed low-turbulence wind tunnel, a high-speed stereo PIV setup with the laser sheet in transverse setup (in *y-z* plane) and two high-speed video cameras (kin cam). For the bats, a feeder system was used to position the animals, while for the birds a perch was used.
(TIF)

Figure S2 **A hypothetical flapping wing that generates tip vortices and a time varying aerodynamic force.** Side view (A) and top view (B) of the flapping wing generating tip vortices with circulation *Γ*(*τ*) and aerodynamic force *F*(*τ*). The lift *L*(*τ*) and thrust *T*(*τ*) components of *F*(*τ*) depend on vortex angle *γ*(*τ*).
(TIF)

Figure S3 **The wake topology for one wingbeat of pied flycatcher #1 flying at 7 m/s.** The wake is visualized as iso-surfaces of streamwise vorticity (blue: ω*_x iso_* = 50 s^−1^; red: ω*_x iso_* = −50 s^−1^) and vertical induced velocities (*w_max_* = 1.7 m/s, see color bar). The different views are (A) perspective view, (B) view from upstream, (C) top view and (D) side view.
(TIF)

Figure S4 **The wake topology for one wingbeat of the blackcap flying at 7 m/s.** The wake is visualized as iso-surfaces of streamwise vorticity (blue: ω*_x iso_* = 70 s^−1^; red: ω*_x iso_* = −70 s^−1^) and vertical induced velocities (*w_max_* = 3.0 m/s, see color bar). The different views are (A) perspective view, (B) view from upstream, (C) top view and (D) side view.
(TIF)

Figure S5 **The wake topology for one wingbeat of the female Pallas' long-tongued bat flying at 7 m/s.** The wake is visualized as iso-surfaces of streamwise vorticity (blue: ω*_x iso_* = 50 s^−1^; red: ω*_x iso_* = −50 s^−1^) and vertical induced velocities (*w_max_* = 2.1 m/s, see color bar). The different views are (A) perspective view, (B) view from upstream, (C) top view and (D) side view.
(TIF)

Figure S6 **The wake topology for one wingbeat of the female lesser long-nosed bat flying at 7 m/s.** The wake visualized as iso-surfaces of streamwise vorticity (blue: ω*_x iso_* = 45 s^−1^; red: ω*_x iso_* = −45 s^−1^) and vertical induced velocities (*w_max_* = 2.4 m/s, see color bar). The different views are (A) perspective view, (B) view from upstream, (C) top view and (D) side view.
(TIF)

Figure S7 **Normalized lift throughout the wingbeat for the main vortex wake structures.** The left panels show the positive normalized lift from tip vortices (solid lines) and tail vortices (dashed lines), at 4 m/s (A) and 7 m/s (B). The right panels show negative normalized lift from root vortices (solid lines) and reversed vortex loops vortices (dashed lines) at 4 m/s (C) and 7 m/s (D). Data are for the pied flycatcher (yellow), blackcap (red), Pallas' long-tongued bat (blue), and lesser long-nosed bat (green). The wingbeat upstroke fractions for the bats and birds are marked with the grey bar at the top and bottom, respectively. Note the differences in scale between the positive and negative lift plots.
(TIF)

Figure S8 **Normalized force productions throughout the normalized flight speed range, for the downstroke and upstroke, respectively.** Force productions consist of lift during the downstroke (A); thrust during the downstroke (B); lift during the upstroke (C); and thrust during the upstroke (D). The data points are for the pied flycatcher (filled diamonds), blackcap (filled squares), Pallas' long-tongued bat (open circles), and lesser long-nosed bat (open triangles). The trend lines are for birds (black dash) and bats (grey dot dash). Note the differences in scale between the plots.
(TIF)

Table S1 **Statistical results for the mixed linear model analysis of normalized lift and thrust production during the downstroke (** ***L/W*** **_down_ and** ***T/W*** **_down_, respectively).** Variables are the degrees-of-freedom (*DoF*), *F*-ratio, the *r^2^*-value, *t*-ratio, and *p*-values. The *p*-values in bold are significant.
(DOC)

Table S2 **Statistical results for the mixed linear model analysis of normalized lift and thrust production during the upstroke (** ***L/W*** **_up_ and** ***T/W*** **_up_, respectively).** Variables are the degrees-of-freedom (*DoF*), *F*-ratio, the *r^2^*-value, *t*-ratio, and *p*-values. The *p*-values in bold are significant.
(DOC)
