# Supplementary figures and images for: Comparing Aerodynamic Efficiency in Birds and Bats Suggests Better Flight Performance in Birds
Source: PLoS One. 2012 May 18;7(5):e37335. doi: 10.1371/journal.pone.0037335 (PMC3356262; doi:10.1371/journal.pone.0037335)

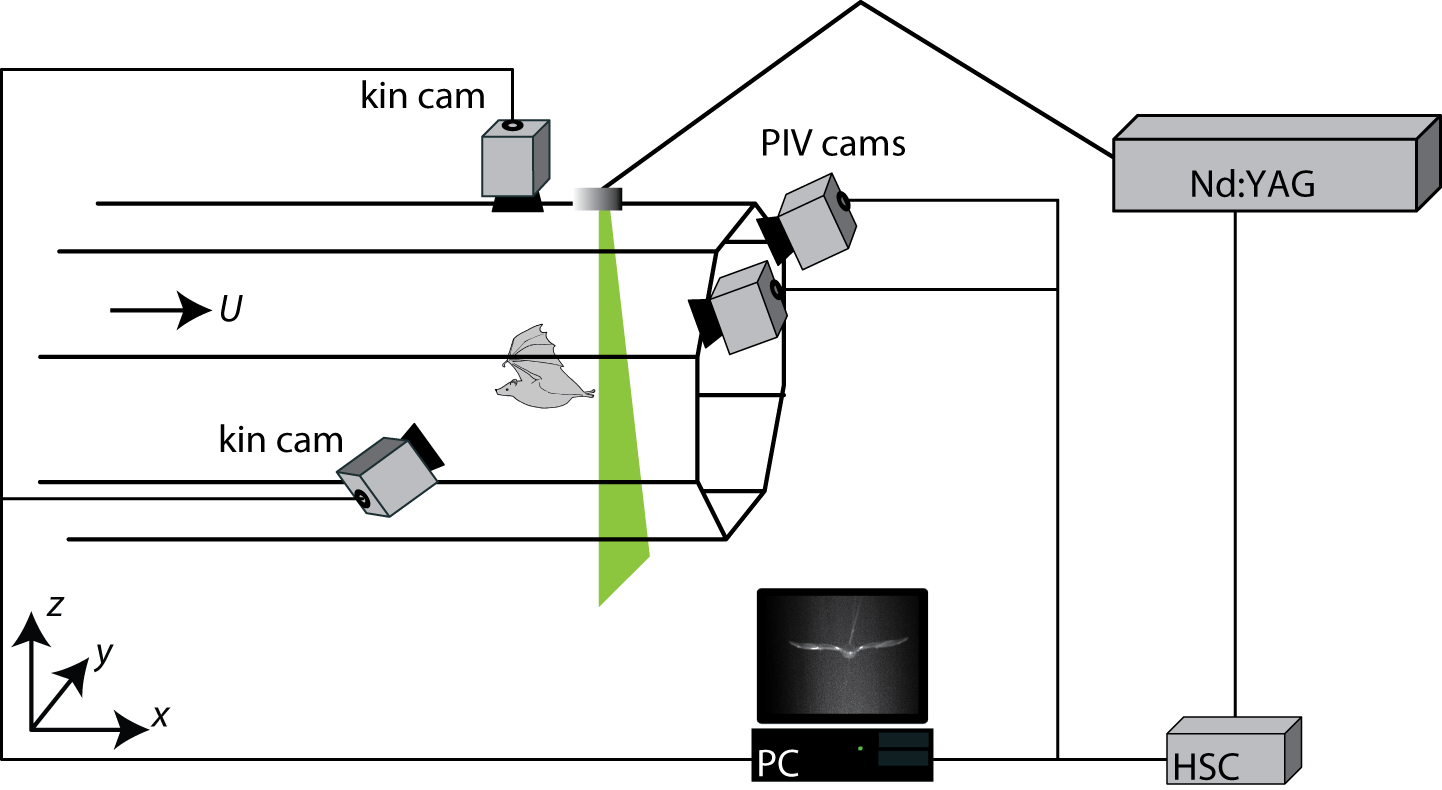

Supplement: Figure S1 — The experimental setup. It consists of a low-speed low-turbulence wind tunnel, a high-speed stereo PIV setup with the laser sheet in transverse setup (in y-z plane) and two high-speed video cameras (kin cam). For the bats, a feeder system was used to position the animals, while for the birds a perch was used. (TIF) [file pone.0037335.s001.tif]

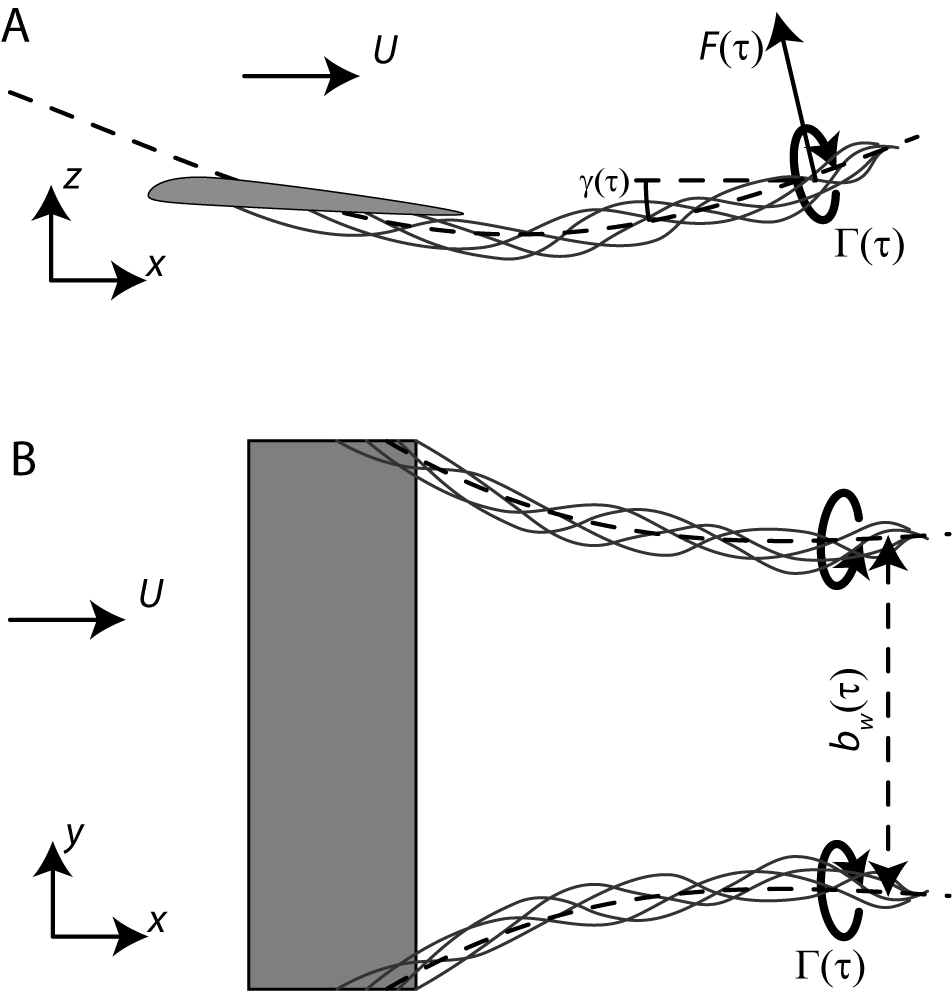

Supplement: Figure S2 — A hypothetical flapping wing that generates tip vortices and a time varying aerodynamic force. Side view (A) and top view (B) of the flapping wing generating tip vortices with circulation Γ(τ) and aerodynamic force F(τ). The lift L(τ) and thrust T(τ) components of F(τ) depend on vortex angle γ(τ). (TIF) [file pone.0037335.s002.tif]

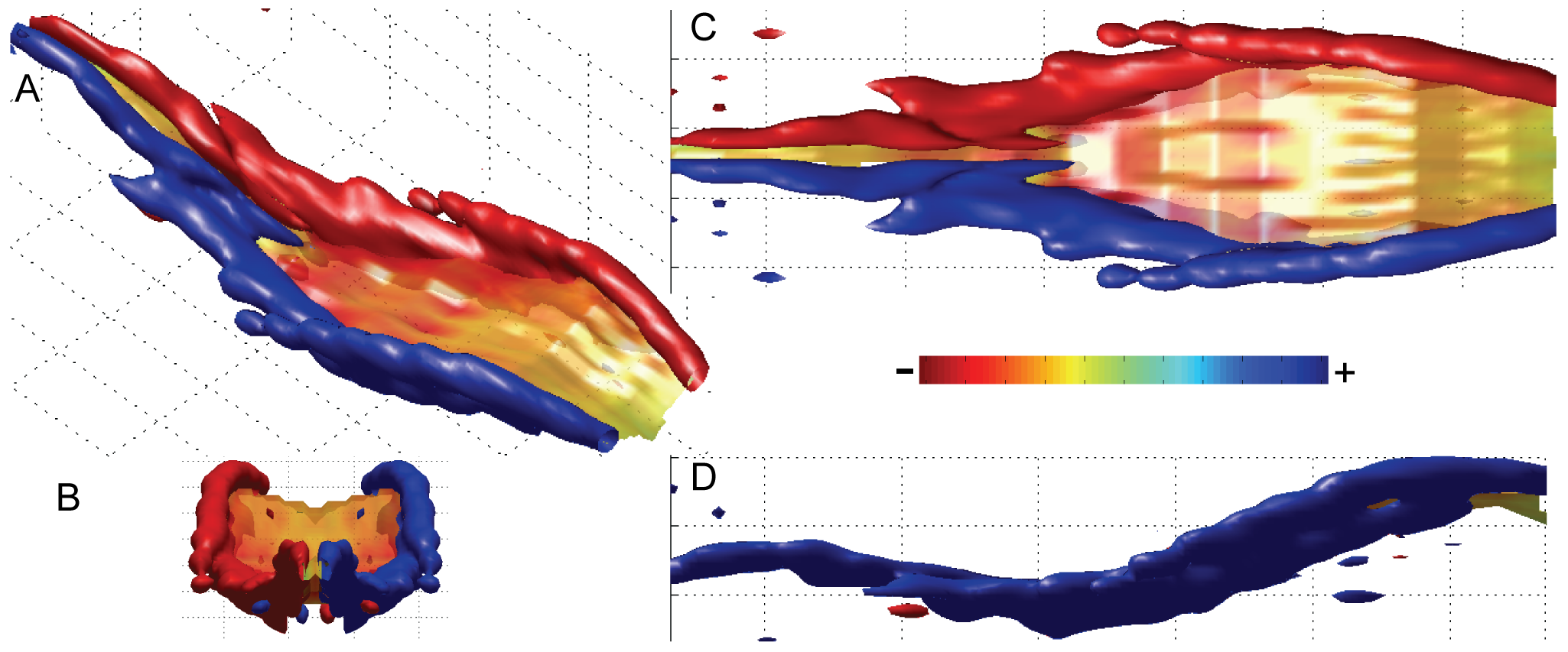

Supplement: Figure S3 — The wake topology for one wingbeat of pied flycatcher #1 flying at 7 m/s. The wake is visualized as iso-surfaces of streamwise vorticity (blue: ωx iso = 50 s−1; red: ωx iso = −50 s−1) and vertical induced velocities (wmax = 1.7 m/s, see color bar). The different views are (A) perspective view, (B) view from upstream, (C) top view and (D) side view. (TIF) [file pone.0037335.s003.tif]

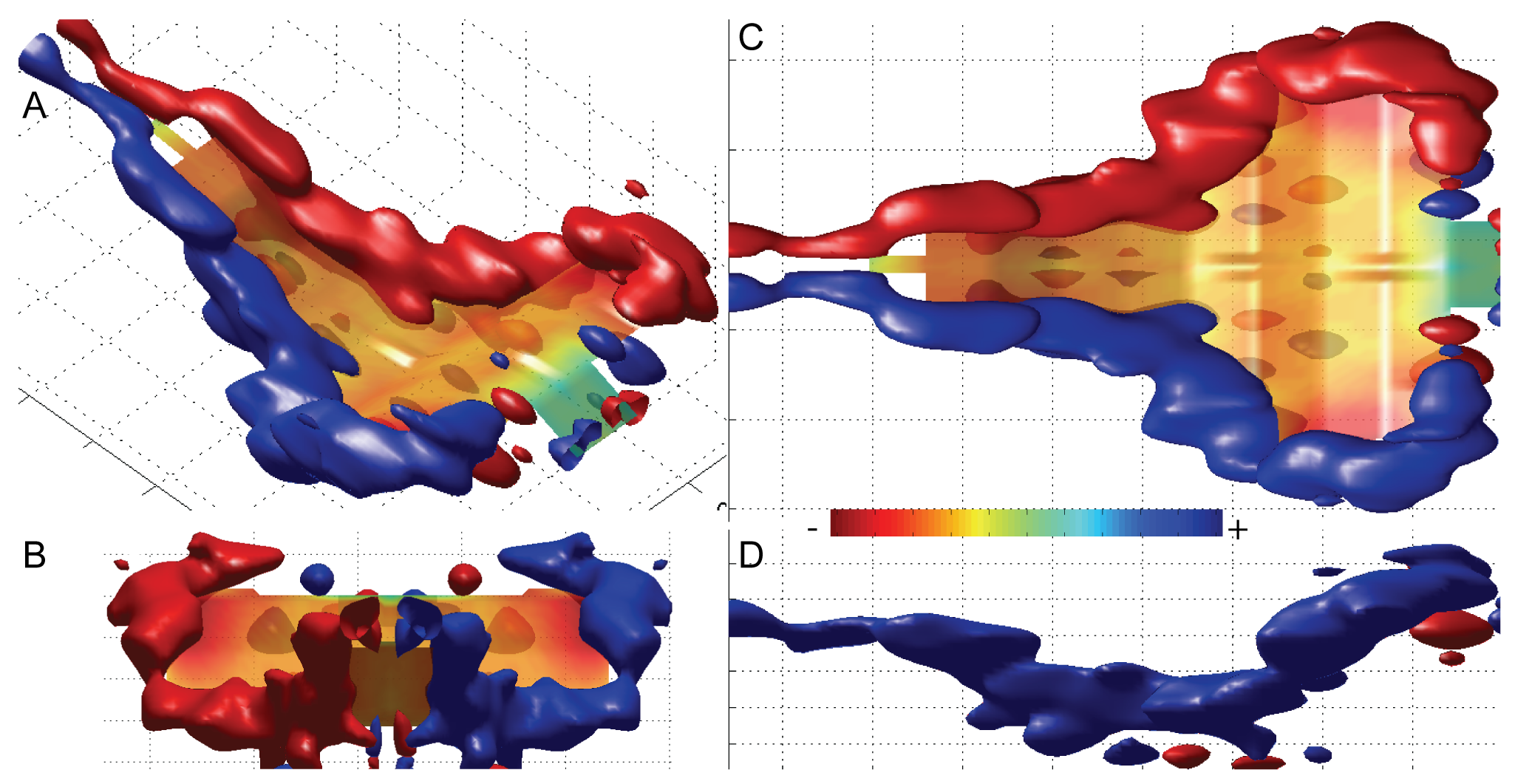

Supplement: Figure S4 — The wake topology for one wingbeat of the blackcap flying at 7 m/s. The wake is visualized as iso-surfaces of streamwise vorticity (blue: ωx iso = 70 s−1; red: ωx iso = −70 s−1) and vertical induced velocities (wmax = 3.0 m/s, see color bar). The different views are (A) perspective view, (B) view from upstream, (C) top view and (D) side view. (TIF) [file pone.0037335.s004.tif]

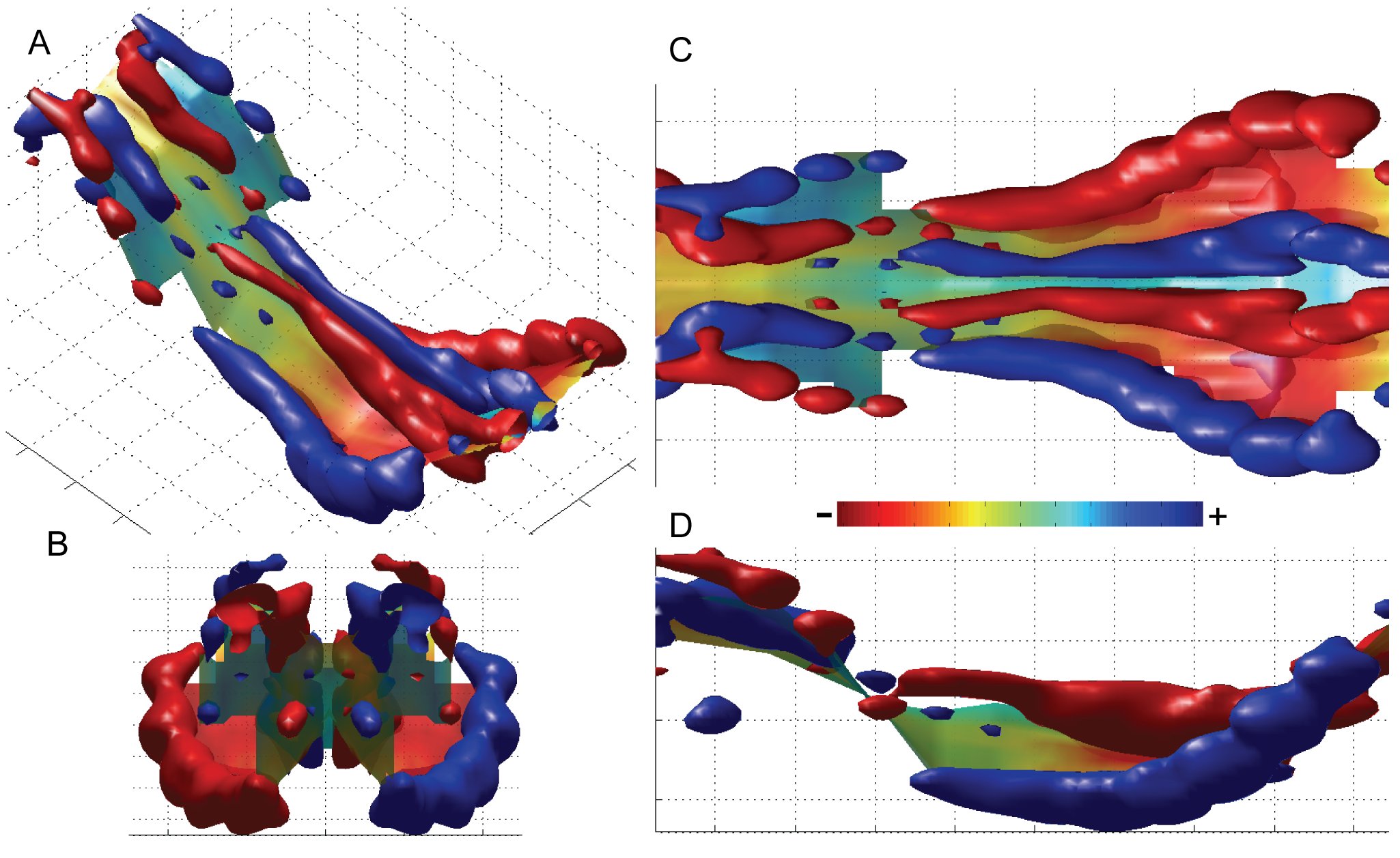

Supplement: Figure S5 — The wake topology for one wingbeat of the female Pallas' long-tongued bat flying at 7 m/s. The wake is visualized as iso-surfaces of streamwise vorticity (blue: ωx iso = 50 s−1; red: ωx iso = −50 s−1) and vertical induced velocities (wmax = 2.1 m/s, see color bar). The different views are (A) perspective view, (B) view from upstream, (C) top view and (D) side view. (TIF) [file pone.0037335.s005.tif]

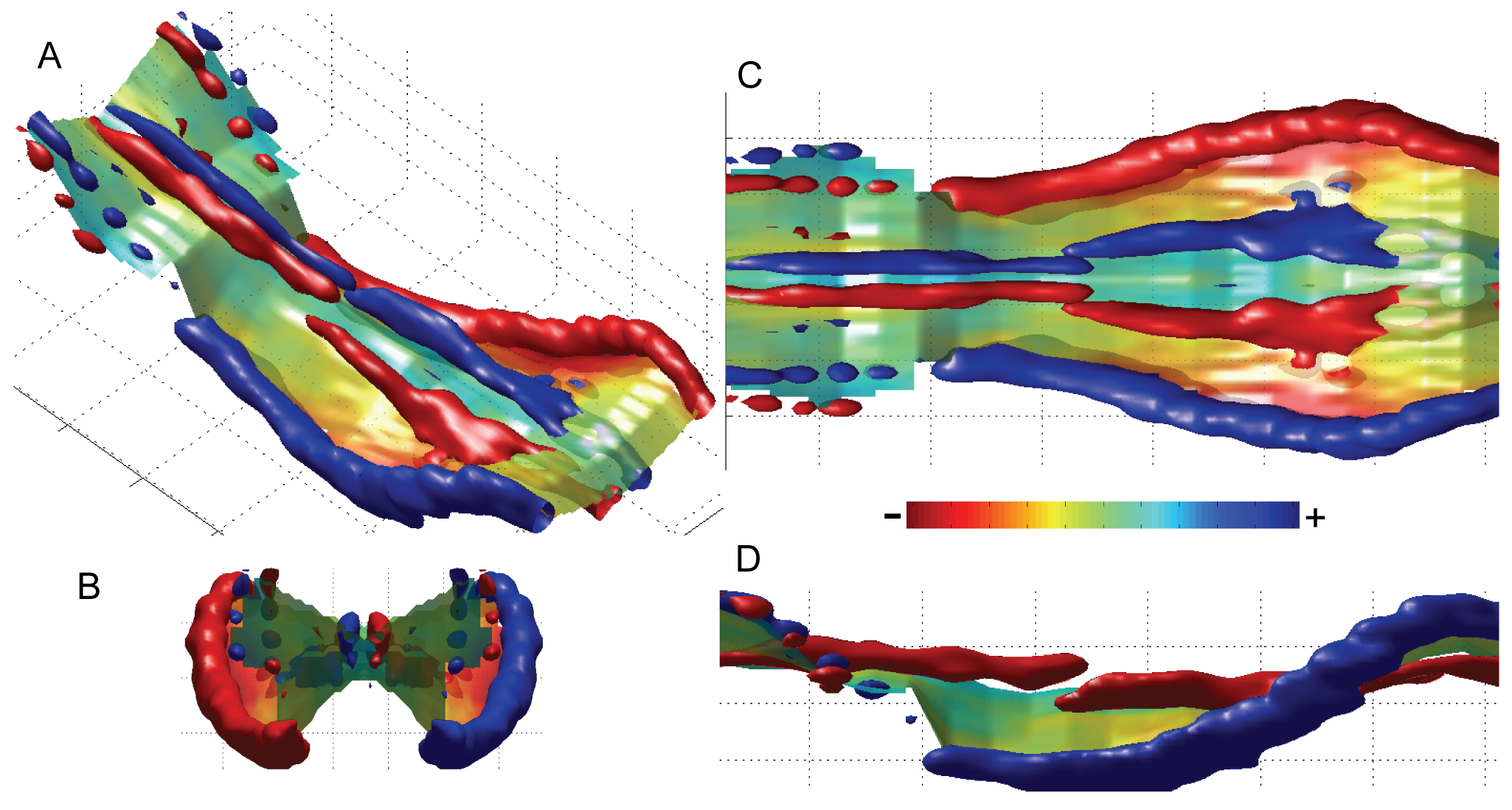

Supplement: Figure S6 — The wake topology for one wingbeat of the female lesser long-nosed bat flying at 7 m/s. The wake visualized as iso-surfaces of streamwise vorticity (blue: ωx iso = 45 s−1; red: ωx iso = −45 s−1) and vertical induced velocities (wmax = 2.4 m/s, see color bar). The different views are (A) perspective view, (B) view from upstream, (C) top view and (D) side view. (TIF) [file pone.0037335.s006.tif]

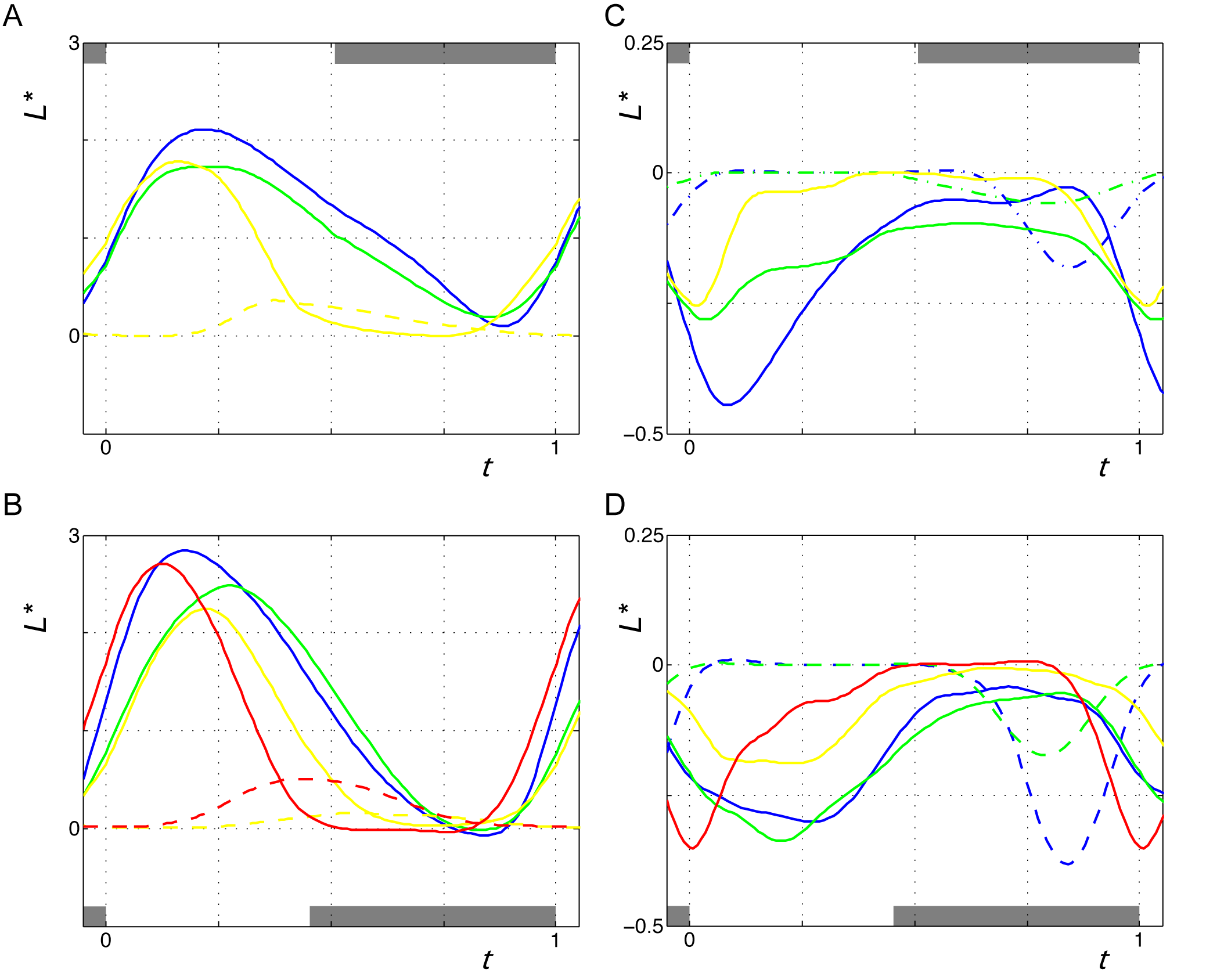

Supplement: Figure S7 — Normalized lift throughout the wingbeat for the main vortex wake structures. The left panels show the positive normalized lift from tip vortices (solid lines) and tail vortices (dashed lines), at 4 m/s (A) and 7 m/s (B). The right panels show negative normalized lift from root vortices (solid lines) and reversed vortex loops vortices (dashed lines) at 4 m/s (C) and 7 m/s (D). Data are for the pied flycatcher (yellow), blackcap (red), Pallas' long-tongued bat (blue), and lesser long-nosed bat (green). The wingbeat upstroke fractions for the bats and birds are marked with the grey bar at the top and bottom, respectively. Note the differences in scale between the positive and negative lift plots. (TIF) [file pone.0037335.s007.tif]

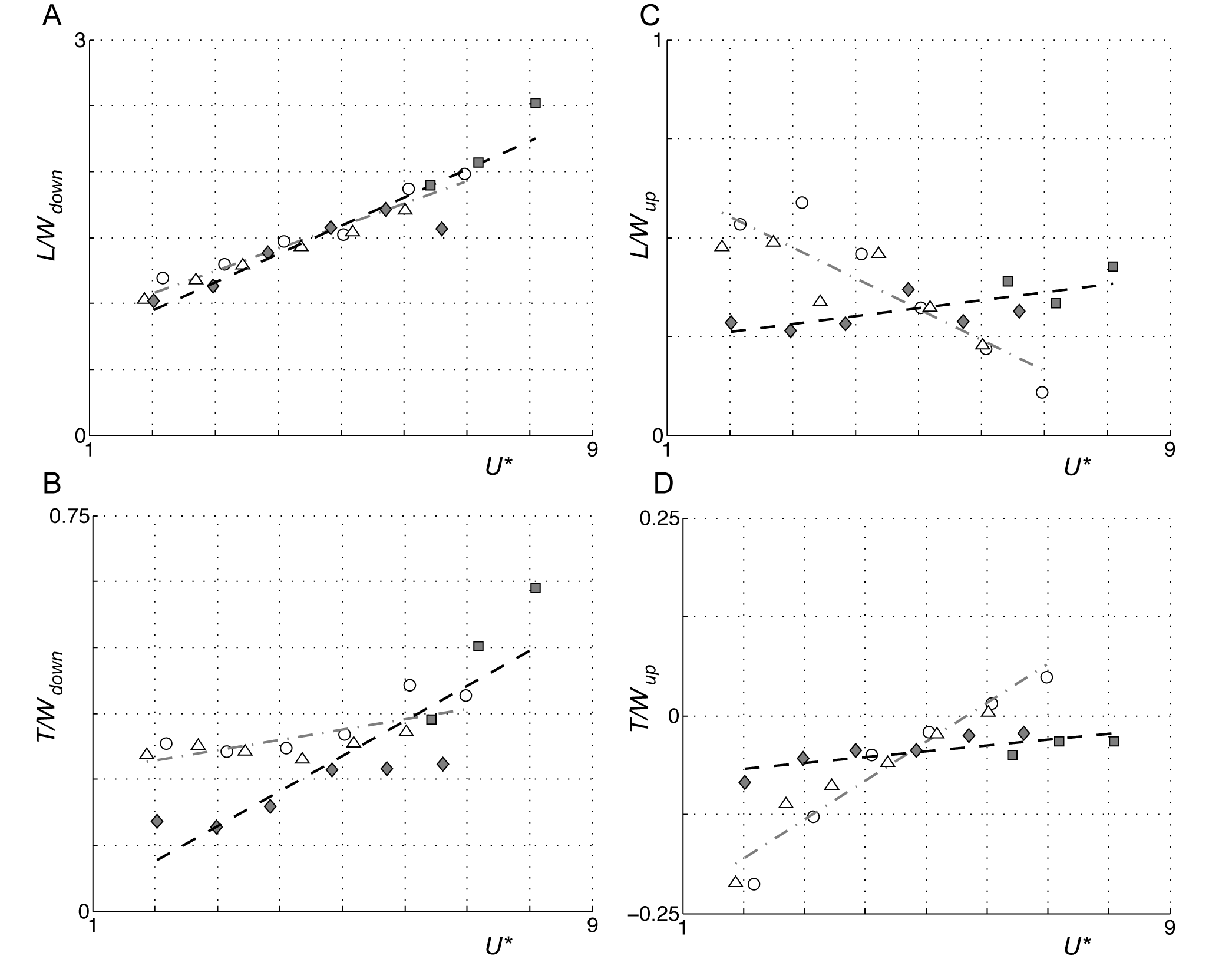

Supplement: Figure S8 — Normalized force productions throughout the normalized flight speed range, for the downstroke and upstroke, respectively. Force productions consist of lift during the downstroke (A); thrust during the downstroke (B); lift during the upstroke (C); and thrust during the upstroke (D). The data points are for the pied flycatcher (filled diamonds), blackcap (filled squares), Pallas' long-tongued bat (open circles), and lesser long-nosed bat (open triangles). The trend lines are for birds (black dash) and bats (grey dot dash). Note the differences in scale between the plots. (TIF) [file pone.0037335.s008.tif]
