# Supplementary material for: Comparing Aerodynamic Efficiency in Birds and Bats Suggests Better Flight Performance in Birds
Source: PLoS One. 2012 May 18;7(5):e37335. doi: 10.1371/journal.pone.0037335 (PMC3356262; doi:10.1371/journal.pone.0037335)
Supplement: Table S1 — Statistical results for the mixed linear model analysis of normalized lift and thrust production during the downstroke ( L/W down and T/W down, respectively). Variables are the degrees-of-freedom (DoF), F-ratio, the r2-value, t-ratio, and p-values. The p-values in bold are significant. (DOC) [file pone.0037335.s009.doc]

**Table S1 statistical results for the mixed linear model analysis of normalized lift and thrust production during the downstroke (*L/W*_down_ and *T/W*_down_, respectively). Variables are the degrees-of-freedom (*DoF*), *F*-ratio, the *r^2^*-value, *t*-ratio, and *p*-values. The *p*-values in bold are significant.**

|  |  | L/W_down_ |  |  | T/W_down_ |  |
| --- | --- | --- | --- | --- | --- | --- |
|  | DF | F-ratio | r^2^ | DF | F-ratio | r^2^ |
| Overall Model | 5 | 50.64 | 0.9441 | 5 | 19.87 | 0.8688 |
|  |  |  |  |  |  |  |
|  | DF | t-ratio | p-value | DF | t-ratio | p-value |
| Intercept | - | 8.98 | **<0.0001** | - | -5.18 | **0.0001** |
| Bird/Bat | 1 | -1.27 | 0.2230 | 1 | -0.96 | 0.3517 |
| U | 1 | 10.27 | **<0.0001** | 1 | -4.28 | **0.0007** |
| U x Bird/Bat | 1 | 0.01 | 0.9954 | 1 | 1.62 | 0.1266 |
